# Screening of Active Compounds Against Porcine Epidemic Diarrhea Virus in *Hypericum japonicum* Thunb. ex Murray Extracts

**DOI:** 10.3390/v17070900

**Published:** 2025-06-26

**Authors:** Hongyu Rao, Siqi Liu, Hao Wu, Wenlong Wang, Weiyue Wang, Weiwei Su, Peibo Li

**Affiliations:** Guangdong Provincial Key Laboratory of Plant Stress Biology, State Key Laboratory of Biocontrol, Guangdong Engineering and Technology Research Center for Quality and Efficacy Re-Evaluation of Post Marketed TCM, School of Life Sciences, Sun Yat-sen University, Guangzhou 510275, China; raohy3@mail3.sysu.edu.cn (H.R.); liusq67@mail2.sysu.edu.cn (S.L.); wuhao8@mail.sysu.edu.cn (H.W.); wangwlong5@mail2.sysu.edu.cn (W.W.); wangwy27@mail2.sysu.edu.cn (W.W.); lsssww@mail.sysu.edu.cn (W.S.)

**Keywords:** *Hypericum japonicum* Thunb. ex Murray, porcine epidemic diarrhea virus, antiviral effect, active compound

## Abstract

Porcine epidemic diarrhea (PED) remains a persistent threat to global swine production, necessitating urgent development of targeted interventions. Our previous research established that *Hypericum japonicum* Thunb. ex Murray (HJT) extract exhibited significant anti-porcine epidemic diarrhea virus (PEDV) activity both in vivo and in vitro. Nevertheless, the principal bioactive constituents mediating this antiviral activity remained uncharacterized. In this study, it was demonstrated that ethanol eluates with 20% (*v*/*v*) and 60% (*v*/*v*) ethanol exhibited activity against PEDV. Phytochemical characterization revealed 66 distinct compounds, including 36 flavonoids and 13 organic acids identified as possible antiviral constituents. Among these, taxifolin-7-O-rhamnoside and quercetin-7-rhamnoside were identified as the most potent anti-PEDV components. Notably, neither compound exhibited significant antiviral efficacy as monotherapy. However, co-administration produced a reduction in PEDV-G2 titers. This study mechanistically links taxifolin-7-O-rhamnoside and quercetin-7-rhamnoside as core anti-PEDV phytochemicals in HJT extract. These findings support the further development of HJT as a potential therapeutic for PED.

## 1. Introduction

Porcine epidemic diarrhea virus (PEDV), a member of the genus *Alphacoronavirus* (family *Coronaviridae*), imposes substantial economic burdens on global swine production systems. The viral particle comprises four structural proteins; the spike (S), envelope (E), and membrane (M) proteins are asymmetrically distributed on the virion surface, whereas the nucleocapsid (N) protein encapsulates the viral RNA genome in a helical conformation [1]. Notably, the N protein demonstrates the highest expression abundance during PEDV infection, functioning as both a potent immunogen eliciting host immune responses and a critical facilitator of viral replication through RNA binding activity [2]. This multifunctional role establishes the N protein as a key biomarker for evaluating antiviral therapeutics. Clinically, PEDV infection manifests as porcine epidemic diarrhea (PED), characterized by acute onset of watery diarrhea, projectile vomiting, and severe dehydration [3,4]. PEDV infects porcine populations across all breeds and age groups, with neonatal piglets showing particularly elevated morbidity and mortality rates (80–100%), establishing this virus as the predominant etiological agent of diarrheal disease in piglets [5]. The virus demonstrates efficient multimodal transmission via fecal–oral, aerosolized, and vertical pathways, enabling rapid intra- and inter-farm dissemination [6].

Current prevention of PEDV is mainly based on the preparation of vaccination against currently prevalent strains, though antigenic variation and genetic divergence among strains compromise vaccine efficacy [7]. Global surveillance data (Swine Disease Reporting System, August 2024) indicate PEDV detection rates of 5.1% in weaned pigs versus 4.3% in sows and adult swine [8]. Without implemented biosecurity protocols, a U.S. swine production network reported PEDV outbreaks affecting 36.5% of holdings within 21 weeks (2019–2020) [9]. Epidemiological surveys estimate a 44% PEDV prevalence in Chinese swine populations [10]. These epidemiological patterns demonstrate PEDV’s persistent socioeconomic impact on global swine production, emphasizing the imperative for improved intervention strategies.

*Hypericum japonicum* Thunb. ex Murray (HJT), a pharmacologically significant annual herb in traditional medicine systems, contains diverse bioactive constituents including flavonoids, phloroglucinols, xanthones, volatile oil, and metallic elements. These components mediate hepatoprotective, antitumor, antimicrobial, cardioprotective, and immunoregulatory activities [11]. Notably, HJT demonstrates broad-spectrum antiviral potential. Flavonoid-enriched fractions exhibit inhibition against hepatitis A virus in avian models (37.0% suppression) and hepatitis B surface antigen production (67.7% suppression) [12,13]. Moreover, seven meroterpenoids based on sericinic acid isolated from *H. japonicum* showed anti-Epstein–Barr virus activity [14]. In addition, multiple pairs of enantiomers extracted from *H. japonicum* showed good inhibitory activity against Kaposi’s sarcoma-associated herpesvirus [15].

In previous studies, we have demonstrated that HJT exhibits significant anti-PEDV activity both in vivo and in vitro, showing inhibitory effects during the viral replication phase [16]. Additionally, therapeutic administration ameliorated clinical manifestations and restored gut microbiota diversity. These findings position HJT aqueous extract as a promising phytotherapeutic candidate for PEDV intervention. However, the pharmacological substance basis of its efficacy remains unclear, making it difficult to establish corresponding quality standards, which hinders its development and promotion for PED treatment. This study was based on the column separation technique and the IPI-FX viral infection model to search for HJT extraction fractions with strong anti-PEDV effects and then combined with network pharmacology to screen the key compounds from them and carried out in vitro validation, thereby elucidating the pharmacological substance basis of the anti-PEDV activity of HJT extract, providing a foundation for its development and quality research.

## 2. Materials and Methods

### 2.1. Reagents

The HJT used in the experiments was purchased from Wuzhou, Guangxi Province, China, and the plant identification was conducted by Dr. Liao Wenbo (Sun Yat-sen University, China). Dulbecco’s modified eagle medium (DMEM), 0.25% Trypsin solution, phosphate buffer solution (PBS), Penicillin–Streptomycin Solution, and Penicillin–Streptomycin (10,000 U/mL) were purchased from Gibco Life Technologies Corporation (New York, NY, USA). The fetal bovine serum albumin (BSA) was purchased from Guangzhou Cellcook Biotech Co., Ltd. (Guangzhou, China). The fetal bovine serum (FBS) was purchased from Gibco Life Technologies Corporation (New York, NY, USA). The Cell Counting Kit-8 (CCK-8) was purchased from Dojindo China Co., Ltd. (Shanghai, China). The ribavirin was purchased from Shanghai Yuanye Bio-Technology Co., Ltd. (Shanghai, China). The RNA Easy kit was purchased from ZScience Biotechnology Co., Ltd. (Roseville, CA, USA). The RT-PCR kit was purchased from Nanjing Wolase Biotechnology Co., Ltd. (Nanjing, China). The GoTaq^®^ qPCR Master Mix was purchased from Promega Co., Ltd. (Madison, WI, USA). The radio immunoprecipitation assay (RIPA) lysis buffer and 5 × SDS loading buffer were purchased from Shanghai Beyotime Biotechnology Co., Ltd. (Shanghai, China). The protease inhibitors were purchased from Yataihengxin Biotechnology Co., Ltd. (Beijing, China). The sodium dodecyl sulfate-polyacrylamide gel electrophoresis (SDS-PAGE), polyvinylidene fluoride (PVDF) membranes, and enhanced chemiluminescence (ECL) reagents were purchased from Bio-Rad Laboratories Co., Ltd. (Hercules, CA, USA). The tris-buffered saline containing Tween-20 (TBST) was purchased from GenStar Biotechnology Co., Ltd. (Beijing, China). The skim milk was purchased from Becton, Dickinson and Company (Franklin Lakes, NJ, USA). The mouse monoclonal antibody against GAPDH was purchased from Abcam Plc (Boston, MA, USA). The mouse monoclonal antibody against PEDV N protein was purchased from Beijing Biolead Biology Sci. & Tech. Co., Ltd. (Beijing, China). The 4% paraformaldehyde and Triton X-100 were purchased from Dalian Meilun Biotech Co., Ltd. (Dalian, China). The macroporous adsorption resin D101 was purchased from Reagent Expert Yunzuoke Co., Ltd. (Guangzhou, China).

### 2.2. Cell Line and Virus

The Immortal Pig Intestinal (IPI-FX) cells were supplied by Dr. Cao Yongchang (Sun Yat-sen University, China). The cells were cultured in DMEM supplemented with 10% inactivated FBS and 1% Penicillin-Streptomycin Solution at 5% CO_2_ and 37 °C. The PEDV-G2 strain was isolated and provided by Dr. Gong Lang (South China Agricultural University, China). The maintenance medium for PEDV propagation was DMEM supplemented with 7.5 μg/mL trypsin. Working stocks for PEDV strains were prepared as in previous studies [16].

### 2.3. Extraction of Herbal Extract and Chemical Compound

After removing the dirt and other impurities, the dry whole plant of HJT was taken, washed to remove the weeds and soil of the grass, and sliced into pieces of about 1–2 cm. Approximately 500 g of HJT was extracted three times using 6 L of deionized water for 0.5 h per extraction. After a total of three times of extractions, the extracts were combined and concentrated. Subsequently, the resin column was eluted sequentially with deionized water, a 20% (*v*/*v*) ethanol aqueous solution, and a 60% (*v*/*v*) ethanol aqueous solution at a flow rate of 3 bed volume/h, in order of decreasing polarity. Elution with the 60% ethanol aqueous solution was continued until the eluate became colorless, effectively eluting all flavonoid components. Each eluent was passed through the column for 3 column volumes, and the eluents were collected and concentrated. This process yielded 126.97 g of water eluate (T0), 176.39 g of 20% (*v*/*v*) ethanol eluate (T20), and 132.05 g of 60% (*v*/*v*) ethanol eluate (T60). All eluents were stored at 4 °C and diluted with methanol or DMEM before use.

### 2.4. Cytotoxicity Assay

Viability of IPI-FX was determined using commercial CCK-8 according to the manufacturer’s instructions. Briefly, cells were spread on a 96-well plate and cultured to 80% confluence and then incubated with T0, T20, and T60 for 48 h, respectively. Among them, the mock group was incubated with DMEM alone, and the solvent group was incubated with DMEM containing 0.1% DMSO as a ribavirin-treated solvent control group. After the cells were washed twice with 1 × PBS (pH 7.4), 10 μL of CCK 8 reagent and 90 μL of DMEM were mixed and added to each well, then incubated at 37 °C for 1 h. The CCK-8 signal was measured at an absorbance of OD450. The relative viability of the cells was calculated as the percentage of the optical density relative to that of the control sample.

### 2.5. Inhibition of Virus Infection

IPI-FX cells were inoculated into 12-well plates or 24-well plates and pretreated for 1 h with DMEM, ribavirin, or drugs (T0, T20, or T60), all of which were prepared in DMEM at the indicated concentrations. Cells were then infected with PEDV (MOI = 0.1) for 2 h. After infection, the inoculum was removed and replaced with DMEM of the same compounds and concentrations as used in the pretreatment. Each well received only one treatment condition, which was maintained throughout the experiment before and after virus infection [17]. The ribavirin-treated group was designated as the positive control. Ribavirin was dissolved in DMSO at a concentration of 100 mg/mL and subsequently diluted with DMEM medium to yield a ribavirin medium solution containing 100 μg/mL ribavirin. The inhibiting effect of drugs on PEDV replication was analyzed as described previously with some modifications [16]. Briefly, cells were collected at 24 h to determine whether any change occurred in the PEDV nucleocapsid (N) protein by Western blot assay and whether any change occurred in the PEDV N gene by qPCR assay. In addition, the inhibitory effect of drugs on viral proliferation was determined by TCID_50_ analysis [18].

### 2.6. qRT-PCR Analysis

Total RNA was extracted from IPI-FX cells using the RNA Easy kit. The RNA concentration was determined using a NanoPhotometer-N60 (Implen, Munich, Germany), and then total RNA (approximately 200 ng) was reverse-transcribed for cDNA synthesis using the RT-PCR kit. Real-time quantitative PCR (qPCR) was performed using GoTaq^®^ qPCR Master Mix according to the manufacturer’s protocol. Experiments were performed on a LightCycler 480 system (Roche, Mannheim, Germany) under the following conditions: initial denaturation at 95 °C for 10 min, followed by 45 cycles of 95 °C for 10 s, 60 °C for 20 s, and 72 °C for 20 s; the last cycle consisted of 95 °C for 5 s and 65 °C for 1 min [16].

The specific primers for the experiments were synthesized by Shanghai Sangong Biotechnology Co. (Shanghai, China), and the specific primers are listed in Table 1. Fold change in gene expression was calculated using the 2^−ΔΔct^ method, and all PCR reactions were performed in triplicate.

### 2.7. Western Blot Analysis

IPI-FX cells were first washed three times with pre-cooled PBS, and then lysed with RIPA Lysis Buffer supplemented with 1% protease inhibitor and centrifuged. Next, the supernatant was mixed with 5× SDS Sampling Buffer and boiled to obtain protein samples. An equal volume of the sample was separated by 10% SDS-PAGE and transferred to a PVDF membrane. The membrane was then incubated overnight at 4 °C with the following primary antibodies: mouse monoclonal antibody against PEDV N protein and mouse monoclonal antibody against GAPDH (1:10,000). The blot was then washed and incubated with HRP-conjugated goat anti-mouse IgG secondary antibody (1:10,000) for 1 h at room temperature. Protein bands were visualized using ECL reagent and imaged using the Tanon5200 system. Bands were densitometrically analyzed using ImageJ 1.54 software, and all experiments were performed in triplicate to ensure reproducibility.

### 2.8. TCID_50_ Analysis

Cells were seeded into a 96-well plate and cultured for 80% confluence, then rinsed with PBS. A 10-fold serial dilution of the virus sample was then added. Each set of 8 replicate wells was incubated at 37 °C for 4 days. An immunofluorescence assay was performed to visualize and document PEDV-infected cells as follows. Cells were fixed with 4% paraformaldehyde for 15 min, then permeabilized with 0.2% Triton X-100 for 10 min at room temperature. The cells were blocked with 1% BSA for 1 h. The cells were incubated with anti-PEDV N polyclonal antibody (1% BSA preparation) at 37 °C for 1 h (or stained overnight at 4 °C) and, finally, incubated with FITC-labeled goat anti-mouse antibody (1% BSA preparation) at 37 °C for 1 h. Each of the above operations was followed by three washes with PBST. The 96-well plate was placed under a fluorescence microscope (Leica DMi8, Leica Microsystems, Wetzlar, Germany), and then the TCID_50_ of the virus was calculated according to the Reed–Muench method.

### 2.9. UFLC-Q-TOF-MS/MS Analysis of T20 and T60

A total of 3.0 g each of T20 and T60 were accurately weighed and sonicated in 10 mL of methanol for 30 min. The supernatant was filtered with 0.22 μm and then injected into an ultra-fast liquid chromatography/quadrupole-time-of-flight tandem mass spectrometry system for analysis (UFLC-Q-TOF-MS/MS). The column was an Acclaim™ Polar Advantage II C18 (4.6 × 250 mm, 5 μm, Thermo Scientific, Waltham, MA, USA), which was maintained at 30 °C. The mobile phases were composed of water with 0.1% formic acid (A) and methanol (B) using a gradient elution of 95–40% A at 0–55 min, 40–10% A at 55–60 min, and 10% A at 60–63 min. The flow rate into the mass spectrum adjusted by the diverter valve was about 0.3 mL/min. Other MS parameters were adopted to be the same as published work [19]. The sample volume injected was set at 10 μL. All the acquisition and analysis of data were controlled by the PeakView Software TM V. 1.1 (AB SCIEX, Foster City, CA, USA).

### 2.10. Network Pharmacological Analysis

The identified 66 components of T20 and T60 were searched and integrated using the SwissTargetPrediction http://www.swisstargetprediction.ch/ (accessed on 5 June 2023) and CTD https://ctdbase.org/ (accessed on 5 June 2023). In the GeneCards database https://www.genecards.org/ (accessed on 7 June 2023), “porcine epidemic diarrhea virus” was used as a keyword to search, download, and integrate all related genes. The intersection between the T20 and T60 targets and PEDV was used to obtain the therapeutic targets of HJT using the Venn diagram. A network diagram of the active components in T20 and T60 was constructed using Cytoscape 3.7.2 software, and then the network analysis tool in Cytoscape was used to analyze the topological parameters of each network and to evaluate the significance of the nodes according to centrality and node degree. The possible active ingredients in T20 and T60 that play an anti-PEDV role were sorted by degree value.

### 2.11. Determination of the Active Compounds and Contents of HJT Using HPLC

All of the reference standard compounds were purchased from Shanghai Yuanye Bio-Technology Co., Ltd., (Shanghai, China). Protocatechuic acid (10.0 mg), rutin (10.0 mg), isoquercetin (10.0 mg), quercetin (10.0 mg), taxifolin 7-rhamnoside (10.0 mg), taxifolin (10.0 mg), quercitrin (10.0 mg), and quercetin 7-rhamnoside (1.0 mg) were accurately weighed and dissolved in methanol to prepare reference standard solutions, each with a final volume of 10 mL. An HPLC analysis was carried out on an Ultimate 3000 DGLC system (Dionex company, Sunnyvale, CA, USA) equipped with Chromeleon 7.2 data processing software, DGP-3600SD dual-ternary Pump, SRD-3600 degasser, WPS-3000SL Automatic Sampler, TCC-3000RS column oven, and DAD detector. The HPLC system was used under the following chromatographic conditions: mobile phase A: acetonitrile, mobile phase B: 0.1% glacial acetic acid solution with pH 4.0 adjusted with triethylamine, column: Acclaim™ Polar Advantage II C18 (4.6 mm × 250 mm, 5 μm), gradient elution: 0–10 min, 10–15% A; 10–25 min, 15–23% A; 25–30 min, 23% A; 30–50 min, 23–60% A; and 50–53 min, 60–90% A. The detection wavelengths were 257 nm and 289 nm, while the column temperature was 30 °C.

The eight reference standard solutions were mixed and diluted to prepare a mixed reference solution containing protocatechuic acid (60 μg/mL), taxifolin 7-rhamnoside (150 μg/mL), rutin (100 μg/mL), isoquercetin (130 μg/mL), taxifolin (50 μg/mL), quercitrin (250 μg/mL), quercetin 7-rhamnoside (50 μg/mL), and quercetin (50 μg/mL). Then, the solution (10 μL) was injected into the liquid chromatograph, and peak areas were recorded. A calibration curve was constructed with five concentration levels of standard samples covering the range of 10–1000 μg/mL. The prepared test solution was continuously injected six times to record the chromatogram and evaluate precision.

A total of six samples were prepared for each herb solution, and reproducibility was confirmed using chromatography. The test solutions were analyzed at 0, 4, 12, 24, 36, 48, and 72 h after preparation to assess their stability. Recovery of analyses was assessed at medium concentration levels with 6 replicates. The content was determined by the corresponding peak area of each compound.

### 2.12. Molecular Docking

A molecular docking study was conducted to investigate the binding affinities of taxifolin-7-O-rhamnoside and quercetin-7-rhamnoside, as well as papain-like protease 2 (PLP-2) and 3C-like protease (3CL^pro^) of PEDV. The 2D structures of taxifolin-7-O-rhamnoside and quercetin-7-rhamnoside were obtained from the PubChem database, converted into 3D structures, and optimized for energy minimization using ChemBioOffice Ultra 13.0.2-Chem3D software. The protein structures of PLP-2 and 3CL^pro^ were retrieved from the Protein Data Bank (PDB) database maintained by the RCSB. These structures were processed using the PyMOL 2.5.2 software to remove water and residual molecules. The docking grid points and dimensions were set in AutoDockTools-1.5.7 software, and semi-flexible molecular docking was carried out using Vina v1.1.2 software, where the higher absolute value of the binding energy represents the higher affinity of the receptor and ligand.

### 2.13. Statistical Analysis

Statistical comparisons were performed using GraphPad Prism 9 software. Accordingly, the significance of the differences between the treatment groups and mock group (cell viability, PFU, N mRNA, etc.) was determined by the ANOVA.

## 3. Results

### 3.1. Evaluation of Cytotoxicity of HJT Extract

**Figure A1:**
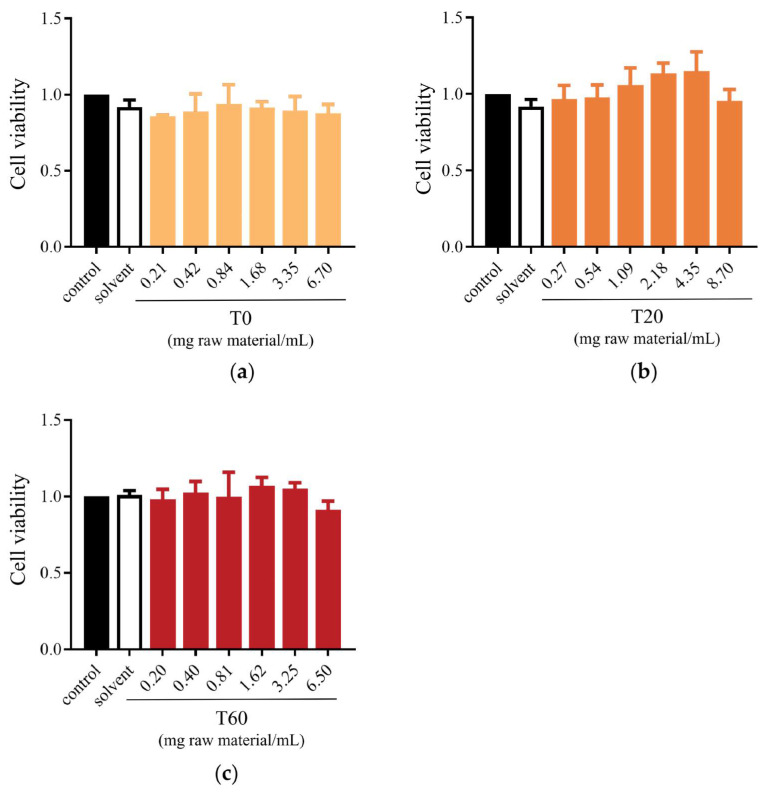
Effects of different elution of HJT extracts on the viability of IPI-FX cells. (**a**) T0 treatment for 48 h, (**b**) T20 treatment for 48 h, (**c**) T60 treatment for 48 h. Data are expressed as mean ± SD, *n* = 3.

CCK-8 was used to detect the effects of T0, T20, and T60 from HJT aqueous extract on the activity of IPI-FX cells for 48 h. The viability of the IPI-FX cells was unaffected by T0, T20, and T60 at concentrations of 0.21–6.7 mg (raw material)/mL, 0.27–8.7 mg (raw material)/mL, and 0.20–6.5 mg (raw material)/mL, respectively (Figure A1). Meanwhile, to exclude the effect of T0, T20, and T60 on pH, a pH meter was used to detect the pH change of DMEM medium after treatment. The results showed that the pH of the drug-containing medium below the maximum safe concentration was not significantly different from that of the negative control. Therefore, the above concentration ranges were selected for the cellular administration treatments without toxic effects on the cells.

### 3.2. Inhibition of Viral Infections In Vitro by HJT Extracts

#### 3.2.1. T0, T20, and T60 Inhibit PEDV N Protein Expression In Vitro

**Figure 1:**
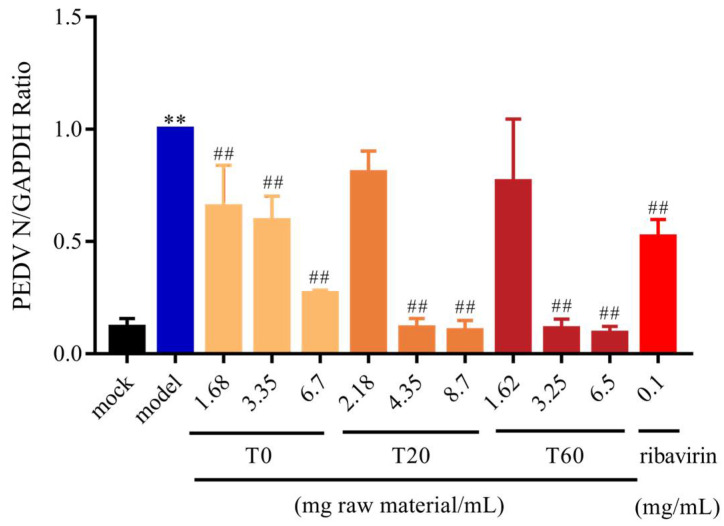
Effect of different elution of HJT extracts on PEDV-G2 N gene. Data are expressed as mean ± SD, *n* = 3; ** *p* < 0.01 compared with the mock group; ^##^ *p* < 0.01 compared with the model group. T0, T20 and T60 treatment groups are indicated by different colours respectively.

**Figure 2:**
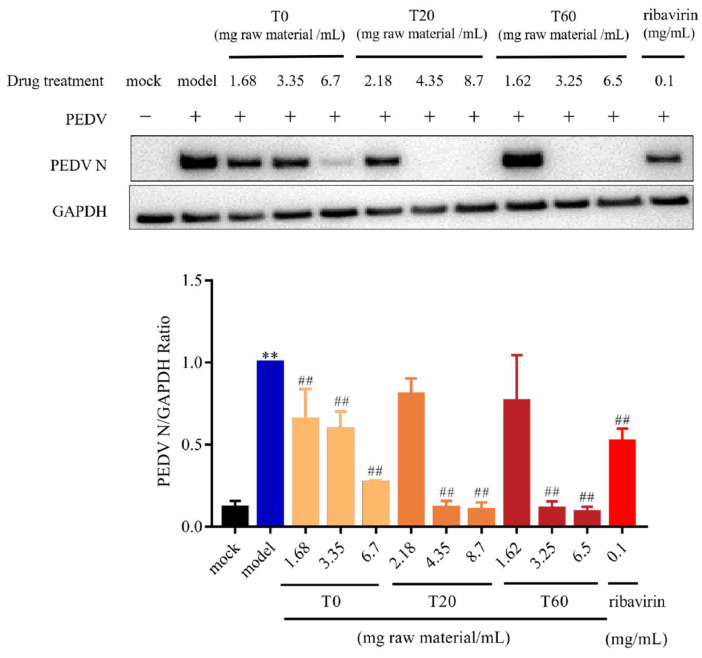
Effect of different elution of HJT extracts on PEDV-G2 N protein. Data are expressed as mean ± SD, *n* = 3; ** *p* < 0.01 compared with the mock group; ^##^ *p* < 0.01 compared with the model group. T0, T20 and T60 treatment groups are indicated by different colours respectively.

qPCR and WB analysis were used to detect the expression of the N protein in IPI-FX after PEDV infection. The results demonstrated that, in comparison with the mock group, the expression level of the N gene in the model group was significantly elevated. In comparison with the model group, N gene expression was found to be significantly reduced in the positive control group after ribavirin treatment. When the concentrations of T0, T20, and T60 reached 3.35 mg (raw material)/mL, 4.35 mg (raw material)/mL, and 1.62 mg (raw material)/mL, respectively, the expression of PEDV N gene was significantly reduced (Figure 1). Further analysis using Western blot showed that the expression of the PEDV N protein was also significantly reduced compared to the model group when the concentrations of T0, T20, and T60 were 1.68 mg (raw material)/mL, 4.35 mg (raw material)/mL, and 3.25 mg (raw material)/mL, respectively (Figure 2, Figure S1). These findings demonstrate that T0, T20, and T60 can significantly decrease the expression of the PEDV-G2 N protein in IPI-FX cells, indicating their in vitro anti-PEDV-G2 activity.

#### 3.2.2. Effect of T0, T20, and T60 on PEDV Titers

**Figure 3:**
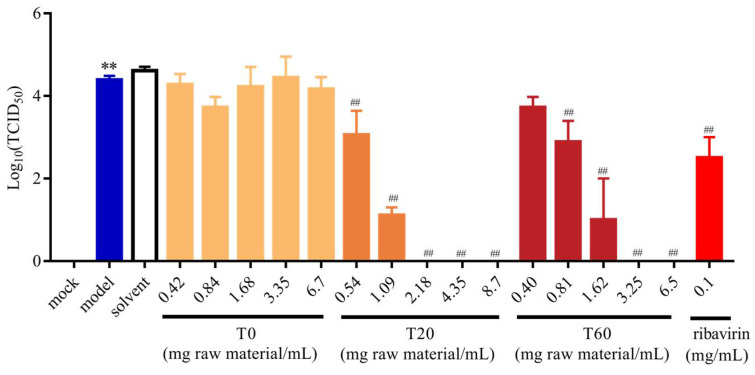
Effect of different elution of HJT extracts on the titers of PEDV-G2. Data are expressed as mean ± SD, *n* = 3; ** *p* < 0.01 compared with the mock group; ^##^ *p* < 0.01 compared with the model group. T0, T20 and T60 treatment groups are indicated by different colours respectively.

For the further evaluation of the anti-PEDV effects of T0, T20, and T60, the changes in PEDV-G2 titers after T0, T20, and T60 treatments were determined using the endpoint dilution method. The results showed that the titers of PEDV-G2 decreased after T20 and T60 treatments, whereas T0 had no significant effect on the titers of PEDV-G2. Meanwhile, ribavirin treatment also significantly decreased the viral titers. In addition, when the concentrations of T20 and T60 reached 2.18 mg (raw material)/mL and 3.25 mg (raw material)/mL, respectively, the viral titers were reduced to 0 (Figure 3). This indicated that both T20 and T60 could make PEDV-G2 lose the ability to reinfect the cells and effectively prevent the transmission of PEDV, whereas T0 was unable to reduce the titers of PEDV. Therefore, it was hypothesized that T20 and T60 were the active fractions in the HJT extracts with anti-PEDV activity.

### 3.3. Anti-PEDV Compounds in HJT Extracts

#### 3.3.1. Characterization of the Chemical Compound Profiles of T20 and T60

**Figure 4:**
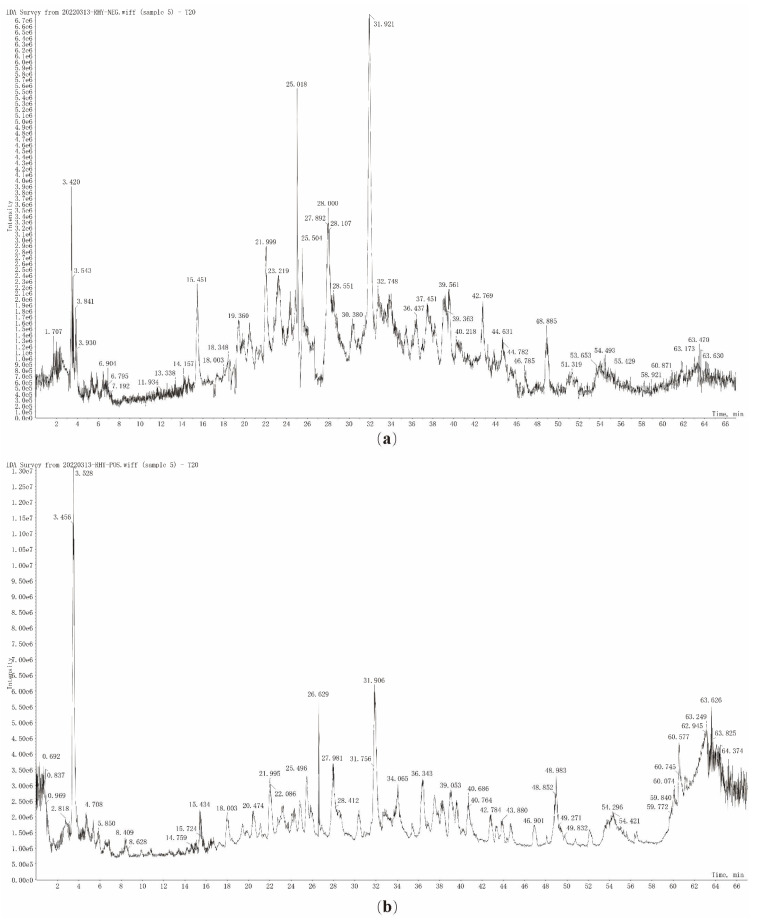
TIC of T20 in the negative ion mode (**a**) and positive ion mode (**b**).

**Figure 5:**
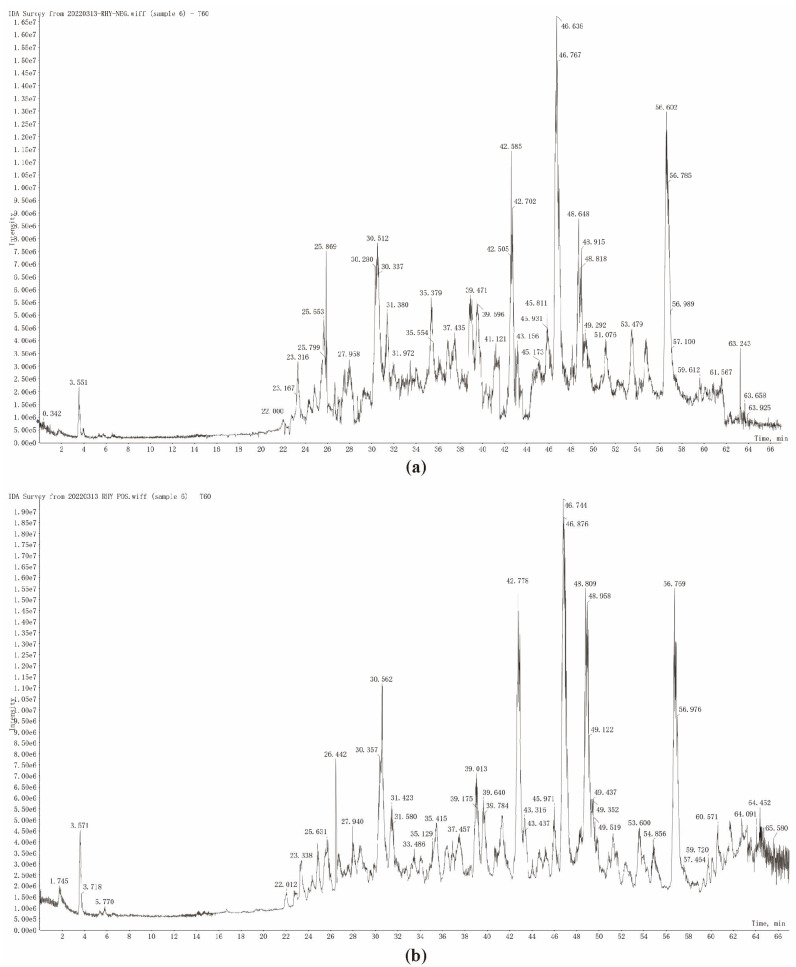
TIC of T60 in the negative ion mode (**a**) and positive ion mode (**b**).

UFLC-Q-TOF-MS/MS analysis was used to detect the chemical compound profile of T20, and its total ion flow diagram was obtained (Figure 4). The mass spectrometry information, such as cleavage fragment ions and retention time of T20, was then compared with the database. In conclusion, 42 compounds were identified, including 26 flavonoids, 5 xanthones, 6 organic acids, and 5 other compounds. The cleavage fragments and peak attribution of each compound in positive and negative ion modes are shown in Table A1. The chemical composition of T60 was analyzed by the same method, and the total ion flow diagrams of T60 were obtained (Figure 5). A total of 46 compounds were identified in T60, including 26 flavonoids, 9 xanthones, 8 organic acids, and 3 other compounds. The cleavage fragments and peak assignments of each compound in positive and negative ion modes are shown in Table A2.

#### 3.3.2. Network Pharmacological Prediction of Anti-PEDV Compounds

**Figure 6:**
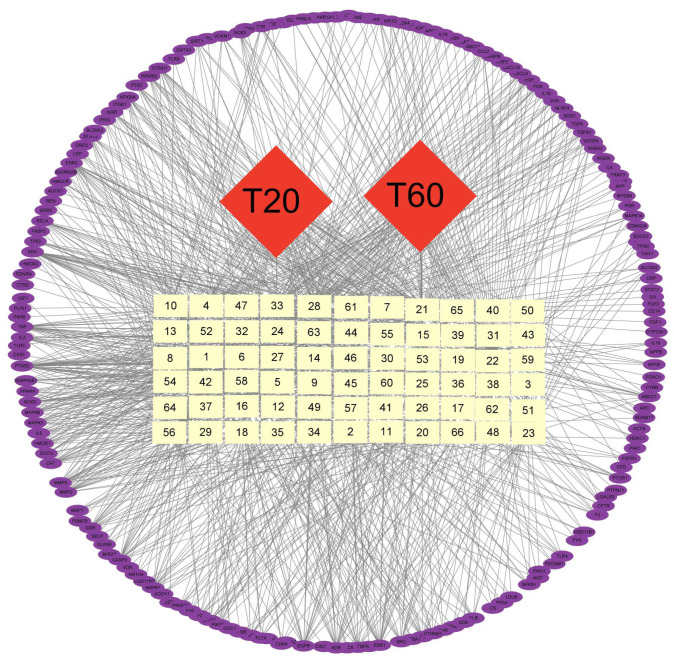
The network of extraction-component-targets. Red quadrilateral, yellow square, and purple circle represent the extraction, the active constituent, and the target, respectively.

We integrated the component analysis results of T20 and T60, identifying a total of 66 compounds. To explore the active ingredients with potential anti-PEDV effects in T20 and T60, the SwissTargetPrediction and CTD databases were used to predict the targets of action of a total of 66 compounds in T20 and T60 (Table A3), and 1228 targets were obtained. After integrating with the 657 PEDV-related targets obtained from the GeneCards database, we obtained 185 potential targets of T20 and T60 against PEDV. The “extraction fraction-component-target” network diagram was constructed and analyzed, and it was found that the target-related compounds were mainly flavonoids and organic acids (Figure 6). Therefore, the anti-PEDV effects of T20 and T60 might be related to the 36 flavonoids and 13 organic acids in the extracts.

#### 3.3.3. Determination of Active Compounds

**Figure 7:**
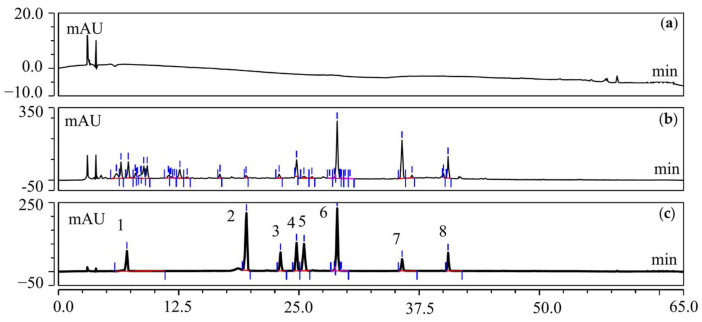
Specificity test results. (**a**) Solvent, (**b**) sample solution: HJT extract, (**c**) mixed substance; peak 1: protocatechuic acid, peak 2: taxifolin 7-rhamnoside, peak 3: rutin, peak 4: isoquercetin, peak 5: taxifolin, peak 6: quercitrin, peak 7: quercetin 7-rhamnoside, peak 8: quercetin.

The contents of the above 36 flavonoids and 13 organic acids were studied by HPLC determination. After mapping the conditions of content determination and methodological investigation, there were eight compounds, including protocatechuic acid, taxifolin 7-rhamnoside, rutin, isoquercitrin, taxifolin, quercitrin, quercetin 7-rhamnoside, and quercetin (Figure 7), that displayed the highest amount in T20 and T60. The content analysis results are shown in Table 2.

### 3.4. Analysis of Anti-PEDV Effects of HJT Active Compounds

#### 3.4.1. Anti-PEDV Effects of Individual Compounds

To investigate whether the anti-PEDV effect of HJT water extract is related to the eight major components mentioned above, this study first examined the effect of each compound on PEDV.

**Figure 8:**
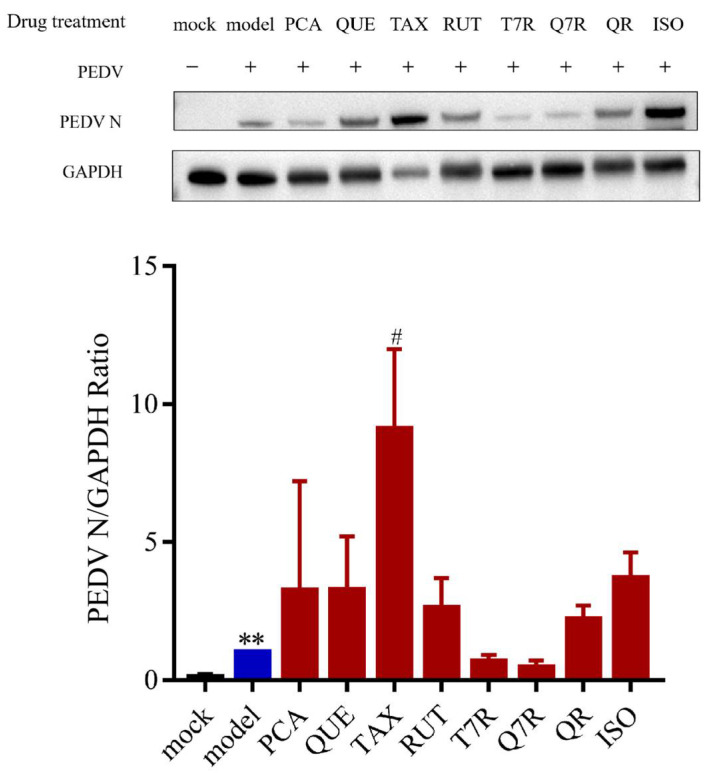
Effect of 8 compounds on PEDV-G2 N protein. PCA: protocatechuic acid; QUE: quercetin; TAX: taxifolin; RUT: rutin; T7R: taxifolin-7-O-rhamnoside; Q7R: quercetin-7-rhamnoside; QR: quercitrin; ISO: isoquercetin. The expression level of N protein was calculated about the expression level of GAPDH. Data are expressed as mean ± SD, *n* = 3; ** *p* < 0.01 compared with the mock group; ^#^ *p* < 0.05, compared with the model group.

Western blot analysis was used to measure the expression levels of PEDV N protein in IPI-FX cells after treatment with the eight compounds. The results showed that, compared to the model group, none of the eight compounds significantly reduced the expression of PEDV N protein (Figure 8, Figure S2). Among them, only the groups treated with taxifolin-7-O-rhamnoside and quercetin-7-rhamnoside showed a trend toward downregulating PEDV N protein expression but without statistical significance. Therefore, it is speculated that the anti-PEDV effect of HJT water extract may be the result of the combined action of multiple compounds, possibly involving taxifolin-7-O-rhamnoside and quercetin-7-rhamnoside.

**Figure 9:**
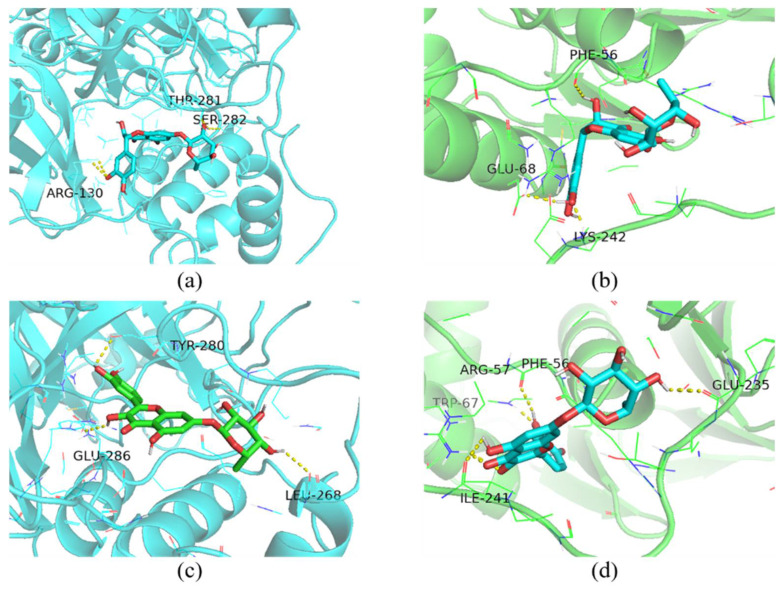
Docking of taxifolin-7-O-rhamnoside and quercetin-7-rhamnoside with PEDV 3CL^pro^ and PLP-2. (**a**) 3CL^pro^ with taxifolin-7-O-rhamnoside, (**b**) PLP-2 with taxifolin-7-O-rhamnoside, (**c**) 3CL^pro^ with quercetin-7-rhamnoside, (**d**) PLP-2 with quercetin-7-rhamnoside. Residues and the ligand are shown as sticks, and hydrogen bonds are represented by black dashed lines.

Furthermore, molecular docking analysis was performed on taxifolin-7-O-rhamnoside and quercetin-7-rhamnoside with key enzymes of PEDV replication (3CL^pro^ and PLP-2) by utilizing the AutoDock-Vina 1.5.7 software (Figure 9). The results demonstrated that the docking binding energies of taxifolin-7-O-rhamnoside with 3CL^pro^ and PLP-2 were −9.5 kcal/mol and −8.0 kcal/mol, respectively, and those of quercetin-7-rhamnoside with 3CL^pro^ and PLP-2 were −10.4 kcal/mol and −8.2 kcal/mol. The specific interacting amino acid residues are shown in Table 3. It has been demonstrated that the two compounds exhibit a strong binding affinity for 3CL^pro^ and PLP-2. The hypothesis is proposed that these compounds may possess an anti-PEDV effect through their interaction with PEDV replication-related proteins, thereby impeding the virus’s replication within host cells.

#### 3.4.2. Anti-PEDV Effects of Mixed Controls

**Figure 10:**
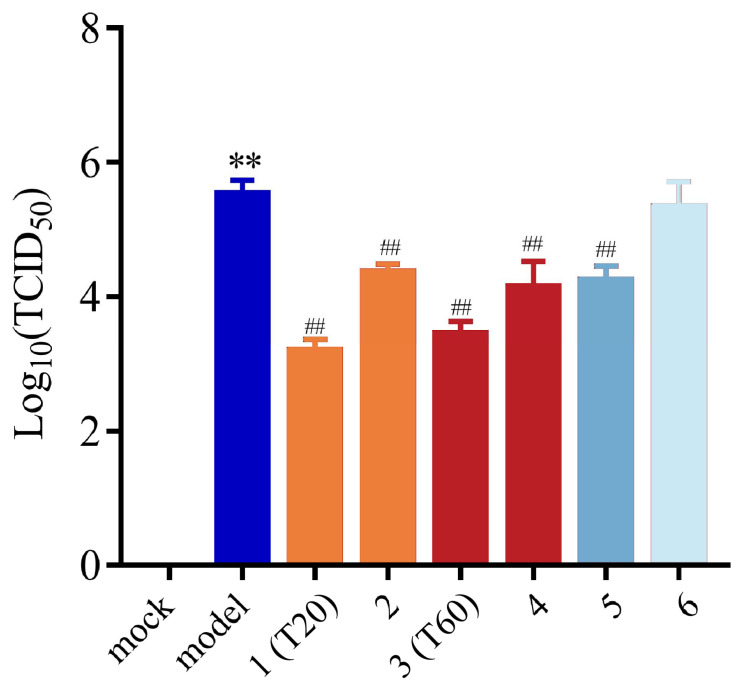
Effect of mixed reference standards on the titers of PEDV-G2. 1: T20 extract; 2: mixture of reference standards of T20 containing 0.11 μg/mL protocatechuic acid, 3.35 μg/mL rutin, 8.45 μg/mL taxifolin-7-O-rhamnoside, 7.17 μg/mL isoquercetin, 1.47 μg/mL taxifolin, 6.81 μg/mL quercitrin, 5.12 μg/mL quercetin-7-rhamnoside, and 0.56 μg/mL quercetin; 3: T60 extract; 4: mixture of reference standards of T60 containing 0.05 μg/mL protocatechuic acid, 0.50 μg/mL rutin, 3.12 μg/mL taxifolin-7-O-rhamnoside, 7.45 μg/mL isoquercetin, 0.86 μg/mL taxifolin, 12.91 μg/mL quercitrin, 4.39 μg/mL quercetin-7-rhamnoside, and 1.62 μg/mL quercetin; 5: mixture of 8.45 μg/mL taxifolin-7-O-rhamnoside and 5.12 μg/mL quercetin-7-rhamnoside; 6: mixture of 0.11 μg/mL protocatechuic acid, 3.35 μg/mL rutin, 7.45 μg/mL isoquercetin, 1.47 μg/mL taxifolin, 12.91 μg/mL quercitrin, and 1.62 μg/mL quercetin. The expression level of N protein was calculated about the expression level of GAPDH. Data are expressed as mean ± SD, *n* = 3; ** *p* < 0.01 compared with the mock group; ^##^ *p* < 0.01 compared with the model group.

Next, we investigated whether it was the mixture reference standards of taxifolin-7-O-rhamnoside and quercetin-7-rhamnoside in the extracts of HJT that exerted the anti-PEDV effect. We prepared mixed controls by referring to the content determination results in 3.2.3 and used Western blot to determine T20 extract (1), a mixture of eight main compounds in T20 (2), T60 extract (3), a mixture of eight main compounds in T20 (4), a mixture of taxifolin-7-O-rhamnoside and quercetin-7-rhamnoside (5), and a mixture of six other compounds (6) and determined their inhibitory effects on PEDV-G2 in vitro. The effects of the six samples on PEDV titers were assessed using the endpoint dilution method. The results indicated that, compared to the model group, T20, T60, the mixed reference standards of T20 and T60, as well as the mixed reference standards of taxifolin-7-O-rhamnoside and quercetin-7-rhamnoside all significantly reduced the PEDV-G2 titers (Figure 10). This suggests that the mixed reference standards composed of taxifolin-7-O-rhamnoside and quercetin-7-rhamnoside have anti-PEDV effects, and it is speculated that these compounds are the key active ingredients responsible for the anti-PEDV activity in the HJT water extract.

## 4. Discussion

PEDV imposes severe socioeconomic impacts on global swine production systems through its high transmission efficiency and devastating mortality profiles, threatening the sustainability of intensive livestock operations. In recent years, plant natural ingredients have begun to be used in anti-PEDV therapy. For example, the aqueous leaf extract of *Moringa oleifera* can inhibit PEDV infection in vitro by inhibiting oxidative stress and apoptosis during the replication phase of PEDV [20]. Thai medicinal plant mulberry’s (*Morus alba* Linn.) leaf ethanolic aqueous crude extracts also showed promising anti-PEDV efficacy [21]. Nevertheless, phytochemical complexity and undefined bioactive markers pose challenges to quality standardization, process reproducibility, and clinical-grade manufacturing of botanical antivirals. Consequently, systematic characterization of bioactive constituents and mechanistic deconvolution of their antiviral pharmacodynamics are critical prerequisites for translating phytomedicines into clinical practice. N protein is widely used in the molecular biological diagnosis of PEDV and is a common pharmacodynamic indicator for evaluating the efficacy of anti-PEDV in vitro. This study measures the expression levels of the PEDV N gene and titers in IPI-FX cells after treatment with T0, T20, T60, and ribavirin. Ribavirin is a broad-spectrum antiviral drug that has been shown to have good antiviral effects against PEDV [22] and was used as a positive control in this experiment. In this study, we tested the anti-PEDV effect and analyzed the composition of different elution sites of HJT extracts separately and determined that T20 and T60 had good anti-PEDV efficacy. Although the N protein content was significantly reduced after treatment with high concentrations of T0, its viral titer did not change significantly. A decrease in N protein indicates that viral proliferation may be inhibited after treatment with a high concentration of T0, while no decrease in viral titer may indicate that the virus is not significantly deprived of its ability to infect. This also reflects the fact that antiviral activity needs to be tested in conjunction with both tests to reach a conclusion. Further, we preliminarily characterized 66 compounds in T20 and T60, which contained flavonoids, xanthones, organic acids, and so on. The anti-PEDV efficacy of T20 and T60 may be related to their rich compound composition.

Extensive research has demonstrated that plant-derived secondary metabolites exhibit significant pharmacological potential. These natural compounds have emerged as a promising focus for antiviral drug discovery, owing to their diverse biological activities, broad availability, favorable safety profiles, and demonstrated capacity to directly interact with viral particles (such as PEDV, dengue virus, transmissible gastroenteritis virus, etc.) [23,24]. Regarding prophylactic mechanisms, matrine has been shown to effectively block PEDV infection through specific interactions with viral surface spike proteins, thereby inhibiting both viral adsorption and cellular entry processes [25]. The PEDV 3CL^pro^, a crucial enzyme in viral replication, plays a pivotal role in both infection and transmission processes. Notably, phytochemicals including wogonin, tomatidine, chrysin, and naringenin exert dual-phase antiviral effects by targeting replicase proteins such as 3CL^pro^, which ultimately disrupts viral particle assembly during both preventive and post-entry stages [26,27,28]. Andrographolide demonstrates distinct therapeutic potential through its apoptosis-inducing mechanism mediated by JAK2-STAT3 pathway inhibition. This pharmacological action not only suppresses PEDV replication but also significantly ameliorates clinical symptoms and reduces mortality rates in infected piglets [29]. In addition, as supplements, ellagic acid and buddlejasaponin IVb can inhibit the inflammatory response induced by PEDV and the former also has the function of antioxidant damage and modulation of the interferon pathway, which improves intestinal homeostasis in PEDV-infected piglets [30,31]. These findings collectively indicate that phytochemical constituents may exert multi-target antiviral effects throughout various stages of viral pathogenesis. Therefore, it is important to investigate the interaction of key components with disease targets for accurate clinical application of natural compounds. In the present investigation, we employed network pharmacology approaches to predict potential antiviral components in HJT extracts, with particular focus on flavonoid and organic acid derivatives. Through content determination and analysis of the anti-PEDV effects of control compounds, taxifolin-7-O-rhamnoside and quercetin-7-rhamnoside were finally hypothesized to be the key anti-PEDV compounds in the extracts of HJT. Notably, antiviral effects were observed exclusively in the combined application of both compounds, demonstrating a statistically significant reduction in PEDV-G2 titers. Meanwhile, 3CL^pro^ and PLP-2 are indispensable key enzymes in the replication process of coronaviruses such as PEDV. These enzymes can promote viral transmission by resisting host cellular immunity, making them important targets for antiviral drug development [32,33,34]. In this study, we found that both taxifolin-7-rhamnoside and quercetin-7-rhamnoside have a strong affinity for these two proteins through molecular docking results. It is hypothesized that they may inhibit viral replication by targeting the viral proteins, which is also supported by our previous findings.

In this study, we analyzed key compounds against PEDV and identified eight important components in HJT, including protocatechuic acid, taxifolin 7-rhamnoside, rutin, isoquercitrin, taxifolin, quercitrin, quercetin-7-rhamnoside, and quercetin. Among these components, taxifolin-7-O-rhamnoside and quercetin-7-rhamnoside were further validated to play a significant role in combating PEDV. Consistently, existing evidence indicates that quercetin-7-rhamnoside demonstrates notable anti-PEDV activity [35], coupled with specific antioxidant properties and cytopathic effect suppression [36]. Recent studies have revealed that plant extracts exhibit enhanced therapeutic efficacy through multi-component, multi-target mechanisms, highlighting the importance of not overlooking the potential roles of the other six compounds. Prior research has confirmed that inter-component interactions among phytochemicals can amplify antiviral (such as dengue virus serotype 2 and SARS-CoV-2) activity through synergistic or additive mechanisms [37,38]. There is an increasing preference for these phytoconstituents in modulating immune responses and enhancing barrier functions [39]. Importantly, gut microbiota-mediated bioconversion can metabolize ancillary components into bioactive derivatives with therapeutic potential [40]. For instance, protocatechuic acid can regulate gut microbiota, decrease levels of inflammatory factors, and modulate the redistribution of tight junction proteins [41]. Dietary supplementation with rutin in weaned piglets enhances intestinal barrier integrity, improves diarrheal resistance, and exerts combined anti-inflammatory and antioxidant effects [42]. Rutin [43], isoquercitrin [44], and quercitrin [45] undergo gut microbiota-mediated bioconversion to bioactive quercetin metabolites that upregulate tight junction protein expression and enhance intestinal epithelial integrity [46]. Taxifolin, on the other hand, activates the Wnt/β-catenin signaling pathway to stimulate tight junction protein synthesis and intestinal epithelial cell proliferation [47].

In summary, our findings demonstrate that taxifolin-7-O-rhamnoside and quercetin-7-rhamnoside represent promising candidate agents for PEDV intervention, with their quantitative levels serving as quality control biomarkers for standardizing HJT-based antiviral formulations. Nevertheless, the precise molecular mechanisms mediating their anti-PEDV activity require further elucidation. Systematic investigation of their antiviral mechanisms is therefore essential for optimizing the therapeutic development and clinical translation of HJT preparations. Notably, the other six major components in HJT extracts may exert ancillary protective roles, despite lacking direct antiviral efficacy under current experimental conditions.

## 5. Conclusions

In conclusion, we identified taxifolin-7-O-rhamnoside and quercetin-7-rhamnoside in the extracts of HJT with in vitro antiviral activity, which significantly reduced the expression of PEDV N protein and viral titers in IPI-FX cells. This study provides candidate compounds for the development of anti-PEDV drugs.

## Figures and Tables

**Table 1:** Primer sequences used for the qRT-PCR.

Gene	Forward Primer (5′→3′)	Reverse Primer (5′→3′)
PEDV N	GAAAATCCTGACAGGCATAAGCA	TTGCCGCTGTTGTCAGACTT
GAPDH	CCTTCCGTGTCCCTACTGC CAAC	GACGCCTGCTTCAC CACCTTCT

**Table 2:** The concentration of 8 compounds in T20 and T60.

Extraction Fraction	Content (mg/g Raw Material)							
	Protocatechuic Acid	Rutin	Taxifolin 7-Rhamnoside	Isoquercitrin	Taxifolin	Quercitrin	Quercetin 7-Rhamnoside	Quercetin
T20	0.009	0.275	0.694	0.589	0.121	0.560	0.420	0.046
T60	0.008	0.076	0.478	1.140	0.132	1.978	0.673	0.247

**Table 3:** Binding energy and interacting amino acid residues of ligands and proteins of PEDV.

Ligand	Protein	Binding Energy (Kcal/mol)	Amino Acid Residues Contributing to Interactions
taxifolin-7-O-rhamnoside	3CL^pro^	−9.5	ARG-130, THR-281, SER-282
	PLP-2	−8.0	PHE-56, GLU-68, LYS-242
quercetin-7-rhamnoside	3CL^pro^	−10.4	LEU-268, TYR-280, GLU-286
	PLP-2	−8.2	PHE-56, ARG-57, TRP-67, GLU-235, ILE-241

**Figure A2:**
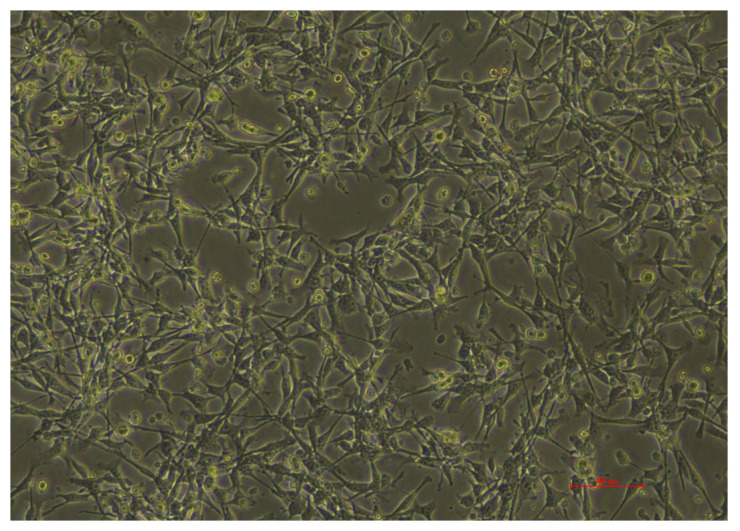
Microscopy of IPI-FX.

## Data Availability

The data can be made available by addressing a request to the corresponding author.

## Supplementary Materials

The following supporting information can be downloaded at: https://www.mdpi.com/article/10.3390/v17070900/s1. Figure S1: WB-original image of Figure 2; Figure S2: WB-original image of Figure 8.

## Abbreviations

The following abbreviations are used in this manuscript:

HJT	*Hypericum japonicum* Thunb. ex Murray
PEDV	Porcine epidemic diarrhea virus
PED	Porcine epidemic diarrhea
DMEM	Dulbecco’s modified Eagle medium
PBS	phosphate buffer solution
BSA	bovine serum albumin
CCK-8	Cell counting Kit-8
RIPA	Radio immunoprecipitation assay
SDS-PAGE	Sodium dodecyl sulfate-polyacrylamide gel electrophoresis
PVDF	polyvinylidene fluoride
ECL	enhanced chemiluminescence
TBST	tris-buffered saline containing tween-20
IPI-FX	immortal pig intestinal
UFLC-Q-TOF-MS/MS	ultra-fast liquid chromatography/quadrupole-time-of-flight tandem mass spectrometry system for analysis.
PLP-2	papain-like protease 2
3CL^pro^	3C-like protease
ARG	Arginine
SER	Serine
THR	Threonine
PHE	Phenylalanine
GLU	Glutamic acid
LYS	Lysine
LEU	Leucine
TYR	Tyrosine
TRP	Tryptophan
ILE	Isoleucine

## Appendix A

**Table A1:** Identification of the chemical profiles of T20 based on UPLC-Triple-TOF-MS/MS.

No.	tR (min)	Molecular Function	Compounds	[M + H]^+^	[M − H]^−^
1	6.65	C_7_H_12_O_6_	Quinic acid	193.0705	191.0562
2	22.03	C_13_H_20_O_3_	Vomifoliol	225.1483	\
3	23.17	C_16_H_18_O_9_	Chlorogenic acid	355.1023	353.0870
4	23.18	C_9_H_6_O_3_	7-hydroxycoumarine	163.0388	\
5	23.26	C_33_H_44_O_8_	2,5-cyclohexadien-1-one, 2-[[3-[(2e)-3,7-dimethyl-2,6-octadien-1-yl]-2,4,6-trihydroxy-5-(2-methyl-1-oxopropyl)phenyl]methyl]-3,5-dihydroxy-4,4-dimethyl-6-(2-methyl-1-oxopropyl)	569.3128	\
6	23.34	C_21_H_22_O_11_	7-o-rhamnoside of flagellin	451.1228	449.1068
7	24.05	C_11_H_12_O_5_	Sinapic acid	225.0755	\
8	24.32	C_15_H_12_O_7_	Taxifolin	305.0655	303.0505
9	24.42	C_28_H_36_O_8_	3,5-dihydroxy-4,4-dimethyl-2-(1-oxoisobutyl)-6-[[5-(1-oxoisobutyl)-3-(3-methyl-2-butenyl)-2,4,6-trihydroxyphenyl]methyl]-2,5-cyclohexadiene-1-one	\	499.2356
10	24.44	C_15_H_14_O_6_	Catechin	291.0862	289.0713
11	25.5	C_26_H_34_O_8_	Saroaspidin c	475.2291	473.2139
12	26.67	C_15_H_14_O_6_	Epicatechin	\	289.0709
13	26.73	C_14_H_10_O_4_	5-hydroxy-1-methoxy-9h-xanthen-9-one	243.0648	\
14	26.74	C_15_H_12_O_6_	5,5′-methylenedisalicylic acid	289.0704	\
15	27.36	C_27_H_30_O_15_	Kaempferol 7-o-rutinoside	595.1644	593.1484
16	27.93	C_27_H_30_O_16_	Quercetin-7-o-rutinoside	611.1595	609.1445
17	27.98	C_18_H_22_O_9_	8-glucosyl-5,7-dihydroxy-2-isopropylchromone	383.1330	381.1178
18	28.01	C_16_H_18_O_8_	3-p-coumaroylquinic acid	\	337.0923
19	30.25	C_27_H_32_O_15_	Ellipticoside	597.1800	595.1643
20	30.57	C_21_H_22_O_11_	Astilbin	\	449.1062
21	31.5	C_19_H_24_O_9_	8-glucosyl-5,7-dihydroxy-2-(1-methylpropyl) chromone	397.1488	395.1330
22	33.06	C_16_H_18_O_8_	4-o-coumaroylquinic acid	\	337.0923
23	33.38	C_27_H_30_O_16_	Rutin	611.1558	609.1442
24	34.87	C_10_H_10_O_4_	Isoferulic acid	\	193.0505
25	34.89	C_10_H_8_O_3_	7-methoxycoumarin	177.0544	\
26	35.43	C_31_H_34_O_8_	4-[[3-benzoyl-2,6-dihydroxy-4-(3-methylbut-2-enoxy)phenyl]methyl]-3,5-dihydroxy-6,6-dimethyl-2-(2-methylpropanoyl)cyclohexa-2,4-dien-1-one	\	533.2121
27	36.5	C_9_H_10_O_4_	Ethyl 3,4-dihydroxybenzoate	183.0650	181.0505
28	36.93	C_27_H_30_O_15_	Quercetin 3,7-di-o-rhamnopyranoside	\	593.1486
29	37.33	C_21_H_20_O_12_	Hyperoside	465.1025	463.0858
30	38.85	C_24_H_20_O_8_	Kielcorin	\	435.1069
31	38.95	C_15_H_12_O_7_	Taxifolin	\	303.0503
32	39.27	C_27_H_30_O_16_	Quercetin 3-o-rutinoside	611.1594	609.1440
33	42.41	C_21_H_20_O_10_	Afzelin	433.1119	431.0962
34	42.76	C_21_H_20_O_12_	Isoquercitrin	465.1020	463.0859
35	43.71	C_12_H_16_O_4_	(1s,3s)-3,4-dihydro-8-methoxy-3,5-dimethyl-1h-2-benzopyran-1,6-diol	225.1099	223.0956
36	46.87	C_21_H_20_O_11_	Quercitrin	449.1067	447.0912
37	48.89	C_21_H_20_O_11_	Vincetoxicoside b	449.1070	447.0913
38	53.85	C_21_H_18_O_13_	Quercetin-3-o-glucuronide	479.0815	\
39	56.88	C_15_H_10_O_7_	Quercetin	303.0499	301.0351
40	57.52	C_13_H_8_O_6_	1,3,5,6-tetrahydroxyxanthone	\	259.0246
41	60.25	C_13_H_8_O_5_	1,3,5-trihydroxyxanthone	\	243.0296
42	63.41	C_28_H_42_O_5_	Hyperjaponol h	\	457.2943

**Table A2:** Identification of the chemical profiles of T60 based on UPLC-Triple-TOF-MS/MS.

NO.	tR (min)	Molecular Formula	Compounds	[M + H]^+^	[M − H]^−^
1	6.61	C_7_H_12_O_6_	Quinic acid	193.0703	191.0563
2	16.62	C_7_H_6_O_5_	Gallic acid	171,0646	169.0144
3	23.14	C_7_H_6_O_4_	Protocatechuic acid	155.0335	153.0195
4	23.2	C_16_H_18_O_9_	Chlorogenic acid	355.1025	353.0876
5	23.2	C_7_H_6_O_3_	4-hydroxybenzoic acid	139.0386	137.0244
6	23.32	C_21_H_22_O_11_	Taxifolin 7-rhamnoside	451.1233	449.1082
7	26.65	C_15_H_14_O_6_	L-epicatechin	291.0864	289.0720
8	27.51	C_45_H_38_O_18_	Procyanidin c1	867.2130	865.1984
9	27.96	C_18_H_22_O_9_	5,7-dihydroxy-2-isopropylchromone-8-beta-d-glucoside	383.1335	381.1190
10	28.23	C_16_H_18_O_8_	3-p-coumaroylquinic acid	\	337.0928
11	30.2	C_27_H_32_O_15_	Ellipticoside	597.1809	595.1664
12	30.48	C_21_H_22_O_11_	Neoisoastilbin	451.1233	449.1084
13	31.47	C_19_H_24_O_9_	8-glucosyl-5,7-dihydroxy-2-(1-methylpropyl) chromone	397.1495	395.1345
14	33.36	C_27_H_30_O_16_	Rutin	611.1601	609.1455
15	34.86	C_10_H_10_O_4_	Isoferulic acid	\	193.0508
16	35.31	C_21_H_22_O_10_	Aromadendrin 7-o-rhamnoside	435.1283	433.1136
17	36.11	C_21_H_20_O_12_	Hyperoside	465.1024	463.0874
18	36.46	C_9_H_10_O4	Ethyl 3,4-dihydroxybenzoate	183.0645	181.0513
19	36.88	C_27_H_30_O_15_	Quercetin 3,7-di-o-rhamnopyranoside	595.1648	593.1501
20	37.11	C_14_H_10_O_6_	1,3,7-trihydroxy-2-methoxyxanthone	275.0548	273.0404
21	37.33	C_14_H_10_O_5_	1,7-dihydroxy-4-methoxyxanthone	259.0600	257.0457
22	37.38	C_9_H_8_O_3_	Trans-4-hydroxycinnamic acid	\	163.0400
23	38.49	C_24_H_20_O_9_	Cadesin a	\	451.1031
24	38.86	C_30_H_24_O_14_	Dehydropolyester catechins	\	607.1087
25	38.88	C_15_H_12_O_7_	Taxifolin	305.0655	303.0511
26	39.04	C_32_H_36_O_8_	Hyperjaponicol a	549.2421	547.2280
27	39.21	C_27_H_30_O_16_	Quercetin 3-o-rutinoside	611.1602	609.1458
28	42.37	C_12_H_18_O_3_	Jasmonic acid	\	209.1182
29	42.45	C_19_H_18_O_10_	Lancerin	407.0971	405.0822
30	42.7	C_21_H_20_O_12_	Isoquercitrin	465.1025	463.0877
31	43.2	C_19_H_28_O_9_	2-cyclohexen-1-one, 4-[(1e)-4-(β-d-glucopyranosyloxy)-3-oxo-1-buten-1-yl]-4-hydroxy-3,5,5-trimethyl-, (4s)-	401.1223	399.1656
32	44.41	C_15_H_12_O_6_	6,8-dihydroxy-1,2-dimethoxy-9h-xanthen-9-one	289.0708	287.0562
33	46.79	C_21_H_20_O_11_	Quercitrin	449.1076	447.0918
34	48.88	C_21_H_20_O_11_	7-(α-l-rhamnopyranosyloxy)-2-(3,4-dihydroxyphenyl)-3,5-dihydroxy-4h-1-benzopyran-4-one	449.1074	447.0917
35	51.53	C_18_H_18_O_8_	Sampsone c	363.1074	361.0916
36	53.53	C_13_H_8_O_6_	Norathyriol	261.0393	259.0248
37	53.84	C_15_H_12_O_5_	Naringenin	273.0756	271.0611
38	54.31	C_21_H_18_O_13_	Quercetin-3-o-glucuronide	479.0816	477.0661
39	54.74	C_21_H_20_O_10_	Kaempferol-7-o-α-l-rhamnoside	433.1129	431.0959
40	55.16	C_13_H_8_O_5_	1,2,5-trihydroxyxanthone	245.0445	243.0299
41	56.57	C_16_H_12_O_7_	Isorhamnetin	317.0657	315.0507
42	56.77	C_15_H_10_O_7_	Quercetin	303.0502	301.0351
43	57.52	C_13_H_8_O_6_	1,3,5,6-tetrahydroxyxanthone	261.0395	259.0246
44	60.06	C_12_H_16_O_4_	3′,3′-dimethyl-6′-oxo-phlorisobutyrophenone	225.1121	223.0977
45	61.62	C_15_H_10_O_6_	Kaempferol	287.0552	285.0403
46	62.84	C_30_H_18_O_10_	3,8′-biapigenin	539.0967	537.0822

**Table A3:** The chemical profiles of T20 and T60.

NO.	Molecular Formula	Compounds	Cas	Categorization
1	C_7_H_12_O_6_	Quinic acid	77-95-2	organic acid
2	C_7_H_6_O_3_	4-hydroxybenzoic acid	99-96-7	organic acid
3	C_7_H_6_O_4_	Protocatechuic acid	99-50-3	organic acid
4	C_7_H_6_O_5_	Gallic acid	149-91-7	organic acid
5	C_7_H_6_O_4_	Ethyl 3,4-dihydroxybenzoate	3943-89-3	organic acid
6	C_9_H_6_O_3_	7-hydroxycoumarin	93-35-6	flavonoid
7	C_9_H_8_O_3_	P-coumaric acid	501-98-4	organic acid
8	C_10_H_10_O_4_	3-hydroxy-4-methoxycinnamic acid	537-73-5	organic acid
9	C_10_H_8_O_3_	7-methoxycoumarin	531-59-9	else
10	C_11_H_12_O_5_	4-hydroxy-3,5-dimethoxycinnamic acid	530-59-6	organic acid
11	C_12_H_16_O_4_	(1s,3s)-3,4-dihydro-8-methoxy-3,5-dimethyl-1h-2-benzopyran-1,6-diol	\	else
12	C_12_H_16_O_4_	3′,3′-dimethyl-6′-oxo-phlorisobutyrophenone	\	xanthone
13	C_12_H_18_O_3_	Jasmonic acid	6894-38-8	organic acid
14	C_13_H_20_O_3_	Vomifoliol	23526-45-6	flavonoid
15	C_13_H_8_O_5_	1,3,5-trihydroxyxanthone	6732-85-0	xanthone
16	C_13_H_8_O_5_	1,2,5-trihydroxyxanthone	156640-23-2	xanthone
17	C_13_H_8_O_6_	1,3,5,6-tetrahydroxyxanthone	5084-31-1	xanthone
18	C_13_H_8_O_6_	Norathyriol	3542-72-1	xanthone
19	C_14_H_10_O_4_	3-methoxy-5-hydroxyxanthenone	\	xanthone
20	C_14_H_10_O_5_	1,7-dihydroxy-4-methoxyxanthone	87339-76-2	xanthone
21	C_14_H_10_O_6_	1,3,7-trihydroxy-2-methoxyxanthone	211948-69-5	xanthone
22	C_15_H_10_O_6_	Kaempferol	520-18-3	flavonoid
23	C_15_H_10_O_7_	Quercetin	117-39-5	flavonoid
24	C_15_H_12_O_5_	Naringenin	480-41-1	flavonoid
25	C_15_H_12_O_6_	5,5′-methylenedisalicylic acid	122-25-8	organic acid
26	C_15_H_12_O_6_	6,8-dihydroxy-1,2-dimethoxy-9h-xanthen-9-one	25991-81-5	xanthone
27	C_15_H_12_O_7_	Epitaxanthin	153666-25-2	flavonoid
28	C_15_H_12_O_7_	Taxifolin	480-18-2	flavonoid
29	C_15_H_14_O_6_	Cianidanol	154-23-4	flavonoid
30	C_15_H_14_O_6_	L-epicatechin	490-46-0	flavonoid
31	C_16_H_12_O_7_	Isorhamnetin	480-19-3	flavonoid
32	C_16_H_18_O_8_	3-p-coumaroylquinic acid	1899-30-5	organic acid
33	C_16_H_18_O_8_	4-p-coumaroylquinic acid	53539-37-0	organic acid
34	C_16_H_18_O_9_	Chlorogenic acid	327-97-9	organic acid
35	C_18_H_18_O_8_	Sampsone c	\	flavonoid
36	C_18_H_22_O_9_	8-glucosyl-5,7-dihydroxy-2-isopropylchromone	188785-44-6	flavonoid
37	C_19_H_18_O_10_	4-β-d-glucosyl-1,3,7-trihydroxyxanthenone	81991-99-3	xanthone
38	C_19_H_24_O_9_	8-glucosyl-5,7-dihydroxy-2-(1-methylpropyl) chromone	188818-27-1	xanthone
39	C_19_H_28_O_9_	2-cyclohexen-1-one,4-[(1e)-4-(β-d-glucopyranosyloxy)-3-oxo-1-buten-1-yl]-4-hydroxy-3,5,5-trimethyl-,(4s)-	189344-54-5	flavonoid
40	C_21_H_18_O_13_	Quercetin-3-o-glucuronide	22688-79-5	flavonoid
41	C_21_H_20_O_10_	Afzelin	482-39-3	flavonoid
42	C_21_H_20_O_10_	Kaempferol 7-o-rhamnoside	20196-89-8	flavonoid
43	C_21_H_20_O_11_	Quercitrin	522-12-3	flavonoid
44	C_21_H_20_O_11_	7-(α-l-rhamnopyranosyloxy)-2-(3,4-dihydroxyphenyl)-3,5-dihydroxy-4h-1-benzopyran-4-one	22007-72-3	flavonoid
45	C_21_H_20_O_12_	Hyperoside	482-36-0	flavonoid
46	C_21_H_20_O_12_	Isoquercitrin	482-35-9	flavonoid
47	C_21_H_22_O_10_	Aromadendrin 7-o-rhamnoside	69135-41-7	flavonoid
48	C_21_H_22_O_11_	Taxifolin 7-rhamnoside	137592-12-2	flavonoid
49	C_21_H_22_O_11_	2-(3,4-dihydroxyphenyl)-5,7-dihydroxy-3-(3,4,5-trihydroxy-6-methyl-oxa n-2-yl)oxy-chroman-4-one	54141-72-9	flavonoid
50	C_24_H_20_O_8_	Kielcorin	64280-48-4	xanthone
51	C_24_H_20_O_9_	Cadesin a	64280-46-2	else
52	C_26_H_34_O_8_	Saroaspidin c	112663-70-4	else
53	C_27_H_30_O_15_	4h-1-benzopyran-4-one, 7-[[6-o-(6-deoxy-α-l-mannopyranosyl)-β-d-glucopyranosyl]oxy]-3,5-dihydroxy-2-(4-hydroxyphenyl)-	103102-81-4	flavonoid
54	C_27_H_30_O_15_	Quercetin 3,7-di-o-rhamnoside	28638-13-3	flavonoid
55	C_27_H_30_O_16_	Quercetin-7-o-rutinoside	147714-62-3	flavonoid
56	C_27_H_30_O_16_	Rutin	153-18-4	flavonoid
57	C_27_H_30_O_16_	Quercetin-3-o-rutinose	949926-49-2	flavonoid
58	C_27_H_32_O_15_	Ellipticoside	128717-89-5	flavonoid
59	C_28_H_36_O_8_	3,5-dihydroxy-4,4-dimethyl-2-(1-oxoisobutyl)-6-[[5-(1-oxoisobutyl)-3-(3-methyl-2-butenyl)-2,4,6-trihydroxyphenyl]methyl]-2,5-cyclohexadiene-1-one	19809-78-0	flavonoid
60	C_28_H_42_O_5_	Hyperjaponol h	\	else
61	C_30_H_18_O_10_	Amentoflavone	101140-06-1	flavonoid
62	C_30_H_24_O_14_	Dehydropolyester catechins	\	flavonoid
63	C_31_H_34_O_8_	4-[[3-benzoyl-2,6-dihydroxy-4-(3-methylbut-2-enoxy)phenyl]methyl]-3,5-dihydroxy-6,6-dimethyl-2-(2-methylpropanoyl)cyclohexa-2,4-dien-1-one	96624-40-7	flavonoid
64	C_32_H_36_O_8_	Hyperjaponicol a	\	flavonoid
65	C_33_H_44_O_8_	2,5-cyclohexadien-1-one, 2-[[3-[(2e)-3,7-dimethyl-2,6-octadien-1-yl]-2,4,6-trihydroxy-5-(2-methyl-1-oxopropyl)phenyl]methyl]-3,5-dihydroxy-4,4-dimethyl-6-(2-methyl-1-oxopropyl)-	105214-57-1	flavonoid
66	C_45_H_38_O_18_	Procyanidin c1	37064-30-5	flavonoid
