# Supplementary figures and images for: Screening of Active Compounds Against Porcine Epidemic Diarrhea Virus in Hypericum japonicum Thunb. ex Murray Extracts
Source: Viruses. 2025 Jun 26;17(7):900. doi: 10.3390/v17070900 (PMC12300913; doi:10.3390/v17070900)

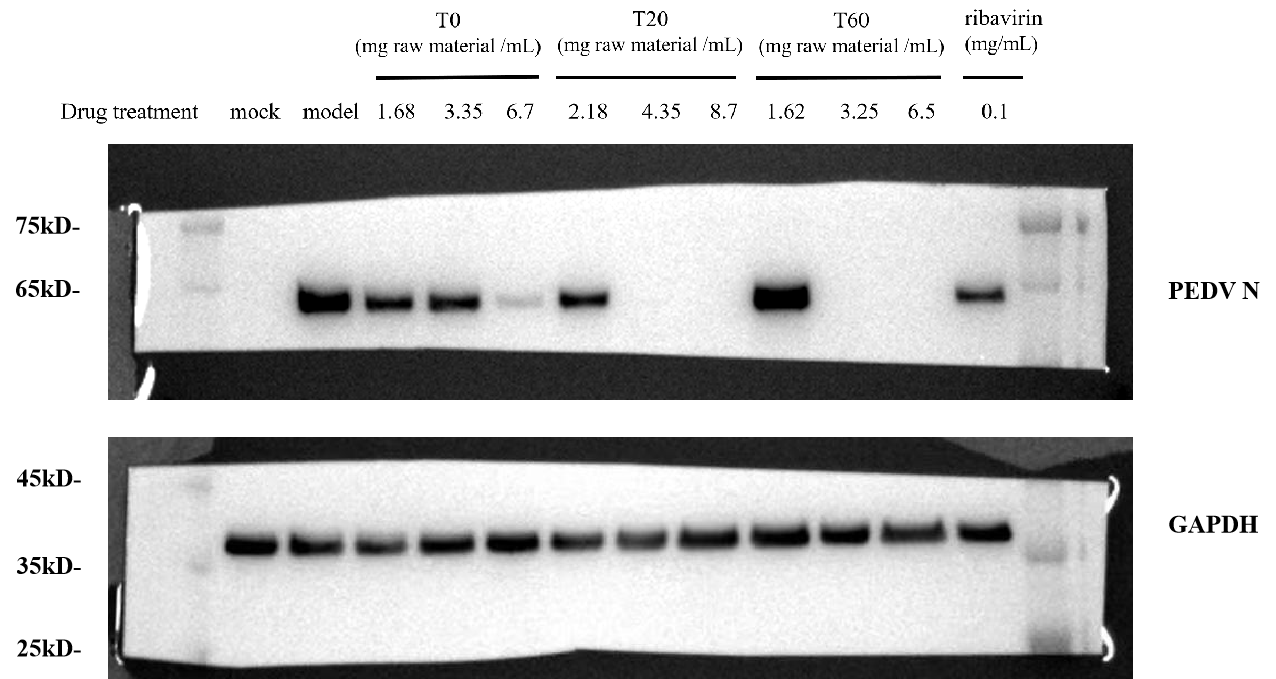

Supplement: Supplementary file 1 [file viruses-17-00900-s001.zip › Figure S1.WB-original image of Figure 2.tif]

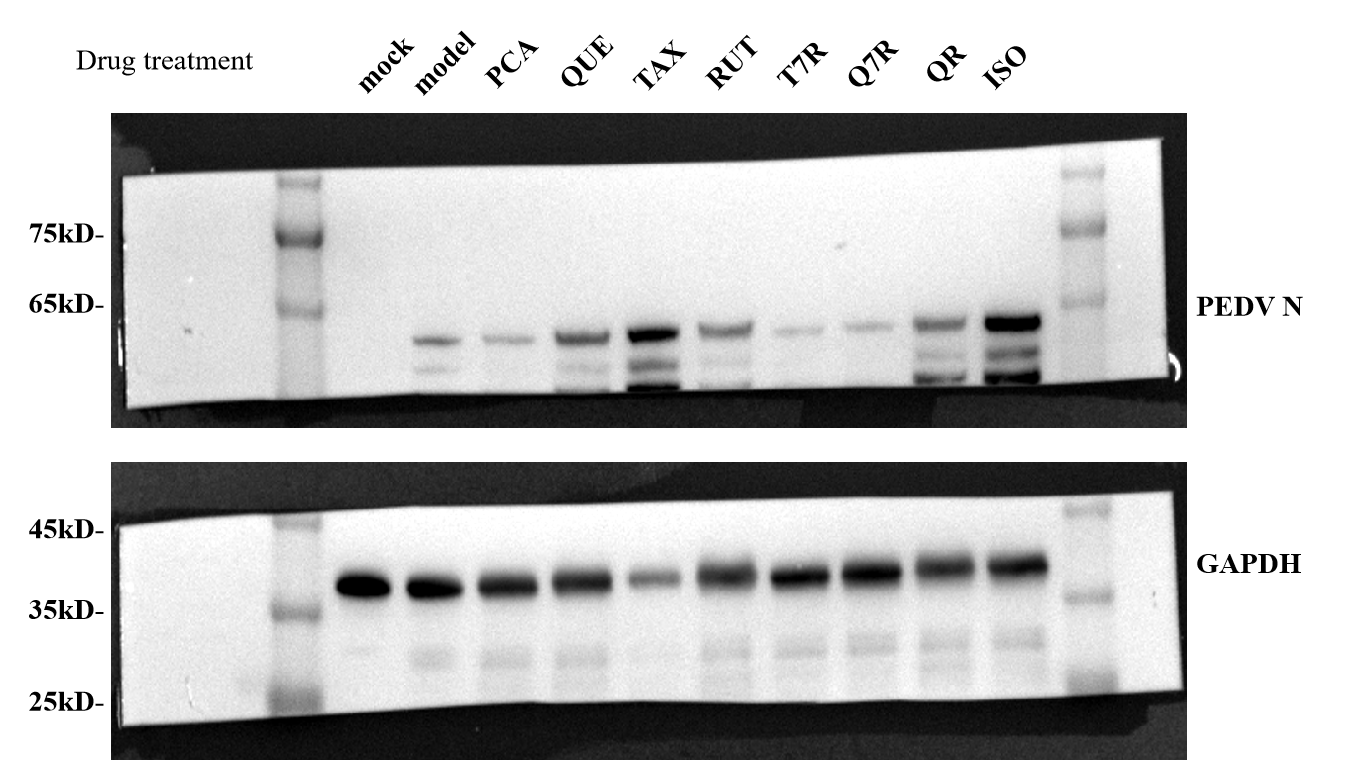

Supplement: Supplementary file 1 [file viruses-17-00900-s001.zip › Figure S2.WB-original image of Figure 8.tif]
